# Vitamin A supplementation coverage and associated factors for children aged 6 to 59 months in integrated and campaign-based delivery systems in four sub-Saharan African countries

**DOI:** 10.1186/s12889-024-18707-3

**Published:** 2024-04-27

**Authors:** Amynah Janmohamed, David Doledec, Romance Dissieka, Umu H. Jalloh, Sugandh Juneja, Maguette Beye, Fatou Ndiaye, Theresia Jumbe, Melissa M. Baker

**Affiliations:** 1 Helen Keller International Vitamin A Supplementation Africa Regional Office, Nairobi, Kenya; 2 Helen Keller International, Freetown, Sierra Leone; 3 Helen Keller International, Dakar, Senegal; 4 Helen Keller International, Dar es Salaam, Tanzania

**Keywords:** Child, Coverage, Delivery strategy, Sub-Saharan Africa, Supplementation, Vitamin A

## Abstract

**Background:**

Vitamin A deficiency (VAD) is a leading contributor to the poor health and nutrition of young children in sub-Saharan Africa. Funding constraints are compelling many countries to shift from longstanding campaigns to integrating vitamin A supplementation (VAS) into routine health services. We assessed child VAS coverage and associated factors for integrated delivery systems in Mozambique, Senegal, and Sierra Leone and for a campaign-based delivery strategy in Tanzania.

**Methods:**

Data were obtained using representative household surveys administered to primary caregivers of *N* = 16,343 children aged 6–59 months (Mozambique: *N* = 1,659; Senegal: *N* = 7,254; Sierra Leone: *N* = 4,149; Tanzania: *N* = 3,281). Single-dose VAS coverage was assessed and bivariate and multivariable associations were examined for child VAS receipt with respect to rural or urban residence; child age and sex; maternal age, education, and VAS program knowledge; and household wealth.

**Results:**

VAS coverage for children aged 6–59 months was 42.8% (95% CI: 40.2, 45.6) in Mozambique, 46.1% (95% CI: 44.9, 47.4) in Senegal, 86.9% (95% CI: 85.8, 87.9) in Sierra Leone, and 42.4% (95% CI: 40.2, 44.6) in Tanzania and was significantly higher for children 6–11 vs. 24–59 months in Mozambique, Senegal, and Tanzania. In Sierra Leone, children aged 12–23 months (aOR = 1.86; 95% CI: 1.20, 2.86) and 24–59 months (aOR = 1.55; 95% CI: 1.07, 2.25) were more likely to receive VAS, compared to those 6–11 months. Maternal awareness of VAS programs was associated with higher uptake in Mozambique (aOR = 4.00; 95% CI: 2.81, 5.68), Senegal (aOR = 2.72; 95% CI: 2.35, 3.15), and Tanzania (aOR = 14.50; 95% CI: 10.98, 19.17). Increased household wealth was associated with a higher likelihood of child VAS in Senegal and Tanzania.

**Conclusions:**

Our findings indicate routine delivery approaches for VAS are not achieving the level of coverage needed for public health impact in these settings. Intensive outreach efforts contributed to the higher coverage in Sierra Leone and highlight the importance of reducing the burdens associated with seeking supplementation at health facilities. As countries move towards incorporating VAS into routine health services, the essentiality of informed communities and potential losses for older children and socio-economically disadvantaged populations are key considerations in the sub-Saharan African context.

## Background

Vitamin A deficiency (VAD) is a leading contributor to the poor health and nutrition of young children in sub-Saharan Africa (SSA) [1]. Global estimates suggest the largest VAD burden exists in SSA, where approximately 50% of children aged 6 to 59 months are affected [2]. Given the importance of maintaining adequate levels of vitamin A during early childhood, and the barriers to children consuming sufficient quantities of vitamin A-rich foods where diets are limited in variety and nutritional quality, twice-yearly vitamin A supplementation (VAS) is recommended for children 6 to 59 months of age in areas where VAD is considered a public health problem [3]. In SSA, VAS has primarily been provided using door-to-door distribution strategies as part of semiannual national child health campaigns (e.g., ‘Child Health Days/Weeks/Months’) which have been highly effective for reaching children across socio-demographic groups [4]. However, fiscal constraints are compelling many countries to reevaluate their delivery mechanisms and, therein, move away from campaign-based approaches and integrate VAS into routine health services [5–8]. This shift aims to ensure continuous availability of VAS at primary level facilities alongside child immunizations, growth monitoring, and other essential child services.

Helen Keller Intl has provided longstanding support for VAS programming in SSA and is assisting governments during the transition to mainstreaming VAS within existing health systems. Routine provision involves the integration of VAS into ongoing services at health facility and community levels and has taken various forms across countries in the region. In Mozambique, VAS delivery platforms have been implemented based on health facility catchment areas, with children from households located < 5 km away supplemented at the health center and those residing 5–10 km and > 10 km away from the health center provided VAS through household visits by community health workers (CHWs) and at fixed site health worker outreach sessions, respectively. In Senegal, VAS is routinely provided at health facilities, in preschools, during community child health checks and growth monitoring activities, and through door-to-door visits by CHWs. As in Mozambique, facility-based supplementation is expected for children from households located within 5 km from health centers. In Sierra Leone, VAS has been integrated into facility-based (fixed and outreach) services, along with periodic intensive routine VAS conducted by health facility staff with the support of CHWs. The outreach component is targeted to reach children 18–59 months due to the low facility attendance for this age group. In Tanzania, VAS continues to be provided through semiannual campaigns held at primary health facilities and community fixed sites during ‘Child Health and Nutrition Months’ occurring in June and December. As an increasing number of countries embark on the transition towards integrating VAS into routine health systems, timely and relevant data are imperative to inform decisions regarding effective implementation models. Therefore, the objective of this study was to assess child VAS coverage and associated factors for integrated delivery systems in Mozambique, Senegal, and Sierra Leone and for a campaign-based delivery strategy in Tanzania.

## Methods

### Study design, sampling, and data collection

Data were obtained using representative household surveys conducted by Helen Keller Intl, in collaboration with national authorities, in 15 districts in Mozambique, 19 districts in Senegal, and two districts in Sierra Leone where VAS was provided on an ongoing basis using routine health facility and community delivery modalities. Surveys were implemented in three regions in Tanzania where a twice-yearly campaign-based VAS delivery system was utilized. Standard survey methodology, including two-stage cluster sampling, was employed in all countries. Each district or region was stratified into rural and urban sampling strata and survey enumeration areas (EAs) were selected using probability proportional to size sampling from cluster lists provided by the National Institute of Statistics or the corresponding entity in each country (Mozambique: 123; Senegal: 444; Sierra Leone: 246; Tanzania: 231). Households were randomly selected from lists created for each EA. Survey eligibility was based on at least one child aged 6 to 59 months residing in the household and a primary child caregiver present at the time of the survey visit.

The enumerators were trained by Helen Keller Intl and administered standardized surveys to primary caregivers in local languages. Information was collected on household, caregiver, and child characteristics; general child health-seeking practices; vitamin A and VAS knowledge and information sources; the child’s VAS status; location of VAS receipt; and reasons for non-supplementation. A household wealth index was constructed using principal components analysis based on asset ownership, housing characteristics, and access to water and sanitation facilities. The child’s age and VAS status were obtained from the caregiver and/or the child’s health card. A sample age-specific vitamin A capsule was shown to assist with caregiver recall. In Mozambique, caregivers were asked whether the child had received VAS during the six months prior to the survey conducted in November 2022. In Senegal and Tanzania, children’s VAS status was obtained for January to June and February to July 2022, respectively. In Sierra Leone, the VAS assessment period was from January to August/September 2022. Survey data were electronically recorded using secured mobile tablets. Though mandatory COVID-19 restrictions had been removed in all four countries by the time of survey implementation, preventive measures including masking, disinfection, and not conducting field work when ill were strongly encouraged. Field supervisors provided oversight for all survey activities. Research ethics approval was obtained from the respective institution in each country and informed consent was required from all survey participants.

### Statistical analysis

The primary study outcome was single-dose VAS coverage, defined as the proportion of children aged 6 to 59 months who received VAS during the specified time period in each country. Bivariate and multivariable associations were assessed for child VAS receipt with respect to: rural or urban residence; child age and sex; maternal age, education, and VAS program knowledge; and household wealth. Model covariates were selected based on a significant (*p* < 0.05) bivariate relationship and/or association with child VAS from prior knowledge. Unadjusted and adjusted odds ratios with 95% confidence intervals are presented in separate analyses for each country as the study was not designed to detect differences in VAS coverage across the four countries. Statistical significance was assessed at *p* < 0.05 for all analyses. Sample-weighted survey data were analyzed using SPSS Complex Samples 26.0 (IBM Corp: Armonk, NY).

## Results

A total of *N* = 16,343 children were included in the study (Mozambique: *N* = 1,659; Senegal: *N* = 7,254; Sierra Leone: *N* = 4,149; Tanzania: *N* = 3,281) (Table 1). The majority of children resided in rural areas across all countries, with approximately two-thirds ≥ 2 years of age. In Mozambique, Senegal, and Tanzania, more than half of children’s mothers were between 20 and 29 years of age and 37.8%, 72.6%, and 13.1% reported having no formal education, respectively. Children from very poor or poor households comprised 5.1% of the study population in Mozambique, 39.6% in Senegal, 30.5% in Sierra Leone, and 44.3% in Tanzania (Table 1).

**Table 1:** Participant characteristics

	Mozambique *N* = 1659	Senegal *N* = 7254	Sierra Leone ^†^ *N* = 4149	Tanzania *N* = 3281
Area				
Rural Urban	1375 (82.9) 284 (17.1)	5824 (80.3) 1430 (19.7)	2801 (67.5) 1348 (32.5)	1798 (54.8) 1483 (45.2)
Child sex				
Female Male	842 (50.8) 817 (49.2)	3493 (48.2) 3761 (51.8)	2042 (49.2) 2107 (50.8)	1565 (47.7) 1716 (52.3)
Child age				
6–11 months 12–23 months 24–59 months	206 (12.4) 380 (22.9) 1073 (64.7)	915 (12.6) 1732 (23.9) 4607 (63.5)	370 (8.9) 995 (24.0) 2784 (67.1)	352 (10.7) 804 (24.5) 2125 (64.8)
Mother’s age				
15–19 years 20–29 years 30–39 years ≥ 40 years	80 (7.6) 637 (60.5) 270 (25.6) 66 (6.3)	353 (5.2) 3631 (53.1) 2402 (35.1) 455 (6.7)	N/A	64 (2.4) 1350 (50.9) 959 (36.1) 280 (10.6)
Mother’s education				
None Primary Secondary or higher	457 (37.8) 539 (44.6) 213 (17.6)	4968 (72.6) 1099 (16.1) 774 (11.3)	N/A	348 (13.1) 1781 (67.1) 525 (19.8)
Household wealth				
Very poor Poor Medium Rich Very rich	84 (5.1) 0 (0.0) 891 (53.7) 338 (20.4) 346 (20.9)	1418 (19.5) 1458 (20.1) 1424 (19.6) 1482 (20.4) 1472 (20.3)	463 (13.2) 610 (17.3) 0 (0.0) 1147 (32.6) 1298 (36.9)	726 (22.1) 727 (22.2) 642 (19.6) 603 (18.4) 583 (17.8)

^†^ Mother’s age and education were not available for Sierra Leone

VAS coverage for children aged 6 to 59 months was below the recommended threshold of 80% [3] in three of four countries: 42.8% (95% CI: 40.2, 45.6) in Mozambique; 46.1% (95% CI: 44.9, 47.4) in Senegal; and 42.4% (95% CI: 40.2, 44.6) in Tanzania (Table 2). VAS coverage exceeded 80% in Sierra Leone (86.9%; 95% CI: 85.8, 87.9). Children in urban, as compared to rural, areas had higher VAS coverage in Mozambique and Senegal. In Mozambique, VAS coverage was 22.3% higher among infants 6–11 months, compared to children 24–59 months (59.2% vs. 36.9%; *p* < 0.001), and was also higher for children aged 12–23 months than for those ≥ 2 years of age (53.7% vs. 36.9%; *p* < 0.001). In Senegal, children 6–11 months had higher VAS coverage compared to those aged 12–23 months (65.1% vs. 55.7%; *p* = 0.001) and 24–59 months (65.1% vs. 39.2%; *p* < 0.001), and VAS coverage was 16.5% higher among children 12–23 months compared to ≥ 24 months (55.7% vs. 39.2%; *p* < 0.001). In Tanzania, higher VAS coverage was also observed for infants, compared to children ≥ 2 years (49.7% vs. 38.1%; *p* = 0.002), and for children 12–23 months compared to 24–59 months (48.3% vs. 38.1%; *p* < 0.001). No significant differences were apparent between child age groups in Sierra Leone. Child sex parity was observed for VAS coverage in all four countries (Table 2).

**Table 2:** Vitamin A supplementation according to socio-demographic characteristics and maternal knowledge

	Mozambique ^1^	Senegal ^2^	Sierra Leone ^3^	Tanzania ^4^
	Child received VAS [*n* (%)]	Child received VAS [*n* (%)]	Child received VAS [*n* (%)]	Child received VAS [*n* (%)]
Area				
Rural Urban	556 (40.4) 166 (58.5) ***	2583 (44.4) 784 (54.8) ***	2424 (86.5) 1177 (87.3)	784 (43.6) 589 (39.7)
Child sex				
Female Male	362 (43.0) 360 (44.1)	1598 (45.7) 1769 (47.0)	1767 (86.5) 1834 (87.0)	651 (41.6) 722 (42.1)
Child age				
6–11 months 12–23 months 24–59 months	122 (59.2) 204 (53.7) 396 (36.9) ***	596 (65.1) **^,^ *** 965 (55.7) *** 1806 (39.2)	310 (83.8) 881 (88.5) 2410 (86.6)	175 (49.7) 388 (48.3) 810 (38.1) **^,^ ***
Mother’s age				
15–19 years 20–29 years 30–39 years ≥ 40 years	37 (46.3) 290 (45.5) 118 (43.7) 31 (47.0)	154 (43.6) 1674 (46.1) 1157 (48.2) 208 (45.7)	N/A	24 (37.5) 589 (43.6) 444 (46.3) 117 (41.8)
Mother’s education				
None Primary Secondary or higher	183 (40.0) 229 (42.5) 117 (54.9) **	2159 (43.5) **^,^ *** 553 (50.3) ** 481 (62.1)	N/A	162 (46.6) 783 (44.0) 229 (43.6)
Household wealth				
Very poor Poor Medium Rich Very rich	40 (47.6) 0 (0.0) 340 (38.2) *^,^ *** 177 (52.4) 165 (47.7)	448 (31.6) *** 654 (44.9) *^,^ *** 696 (48.9) ** 728 (49.1) ** 841 (57.1)	398 (86.0) 535 (87.7) 0 (0.0) 981 (85.5) 1138 (87.7)	312 (43.0) 309 (42.5) 271 (42.2) 250 (41.5) 231 (39.6)
Mother’s VAS knowledge				
Yes No	430 (54.9) *** 99 (23.2)	929 (68.0) *** 2264 (41.4)	2181 (87.1) 461 (89.0)	790 (83.9) *** 384 (22.4)
VAS % (CI)	42.8 (40.2, 45.6)	46.1 (44.9, 47.4)	86.9 (85.8, 87.9)	42.4 (40.2, 44.6)

VAS, vitamin A supplementation; CI, confidence interval. * *p* < 0.05, ** *p* < 0.01, *** *p* < 0.001

^1^ For Mozambique: rural vs. urban (*p* < 0.001), 6–11 months vs. 24–59 months (*p* < 0.001), 12–23 months vs. 24–59 months (*p* < 0.001),

none vs. secondary/higher education (*p* = 0.001), primary vs. secondary/higher education (*p* = 0.005), medium vs. rich (*p* < 0.001),

medium vs. very rich (*p* = 0.012), VAS knowledge vs. no VAS knowledge (*p* < 0.001)

^2^ For Senegal: rural vs. urban (*p* < 0.001), 6–11 months vs. 12–23 months (*p* = 0.001), 6–11 months vs. 24–59 months (*p* < 0.001),

12–23 months vs. 24–59 months (*p* < 0.001), none vs. primary education (*p* = 0.007), none vs. secondary/higher education (*p* < 0.001),

primary vs. secondary/higher education (*p* = 0.002), very poor vs. all wealth quintiles (*p* < 0.001), poor vs. medium (*p* = 0.043), poor vs. rich

(*p* = 0.015), poor vs. very rich (*p* < 0.001), medium vs. very rich (*p* = 0.003), rich vs. very rich (*p* = 0.008), VAS knowledge vs. no VAS

knowledge (*p* < 0.001)

^3^ For Sierra Leone: mother’s age and education were not available; no significant differences in other variables

^4^ For Tanzania: 6–11 months vs. 24–59 months (*p* = 0.002), 12–23 months vs. 24–59 months (*p* < 0.001), VAS knowledge vs. no

VAS knowledge (*p* < 0.001)

In Mozambique, VAS coverage was higher for children of mothers with a secondary or higher education compared to no formal education and compared to those with a primary level education (Table 2). In Senegal, children of mothers with a primary education and with a post-primary education had higher VAS coverage than children of non-educated mothers. Additionally, in Senegal, children in the poorest households had lower VAS coverage compared to children from households in all the other wealth quintiles (*p* < 0.001). Knowledge about the VAS program was reported by 64.8% of mothers in Mozambique, 20.0% in Senegal, 82.9% in Sierra Leone, and 35.5% in Tanzania. Substantially higher VAS coverage was observed for children whose mothers had knowledge of the VAS program, compared to no awareness, in Mozambique (54.9% vs. 23.2%; *p* < 0.001), Senegal (68.0% vs. 41.4%; *p* < 0.001), and Tanzania (83.9% vs. 22.4%; *p* *<* 0.001) (Table 2).

In the multivariable models examining key determinants of child VAS, children in rural areas had a decreased likelihood of receiving VAS in Mozambique compared to their urban counterparts (aOR = 0.52; 95% CI: 0.34, 0.79) (Table 3). In Sierra Leone, children aged 12–23 months (aOR = 1.86; 95% CI: 1.20, 2.86) and 24–59 months (aOR = 1.55; 95% CI: 1.07, 2.25) were more likely to receive VAS compared to those 6–11 months. Conversely, children aged 24–59 months were less likely to receive VAS, as compared to infants 6–11 months, in Mozambique, Senegal, and Tanzania. In Senegal, children of mothers with a secondary or higher education level were more likely to receive VAS, compared to children of non-educated mothers (aOR = 1.75; 95% CI: 1.45, 2.12). In contrast, both primary and secondary or higher maternal education were associated with a reduced likelihood of child VAS in Tanzania. Increased household wealth was associated with VAS uptake in Senegal and Tanzania. Lastly, maternal awareness of the VAS program significantly increased VAS uptake in Mozambique (aOR = 4.00; 95% CI: 2.81, 5.68), Senegal (aOR = 2.72; 95% CI: 2.35, 3.15), and Tanzania (aOR = 14.50; 95% CI: 10.98, 19.17) (Table 3).

**Table 3:** Multivariable associations with vitamin A supplementation

	Mozambique		Senegal		Sierra Leone ^†^		Tanzania	
	UOR	AOR	UOR	AOR	UOR	AOR	UOR	AOR
Area								
Urban Rural	1.00 0.46 (0.33, 0.64)	1.00 0.52 (0.34, 0.79)	1.00 0.64 (0.56, 0.73)	1.00 0.87 (0.74, 1.02)	1.00 0.94 (0.72, 1.23)	1.00 0.92 (0.71, 1.19)	1.00 1.30 (1.09, 1.54)	1.00 1.26 (0.94, 1.70)
Child sex								
Male Female	1.00 0.92 (0.71, 1.20)	1.00 0.90 (0.67, 1.22)	1.00 0.96 (0.87, 1.07)	1.00 0.95 (0.85, 1.06)	1.00 1.01 (0.79,1.29)	1.00 1.01 (0.79, 1.29)	1.00 0.89 (0.73, 1.09)	1.00 0.86 (0.67, 1.10)
Child age								
6–11 months 12–23 months 24–59 months	1.00 0.83 (0.53, 1.31) 0.40 (0.27, 0.60)	1.00 0.78 (0.47, 1.30) 0.33 (0.21, 0.52)	1.00 0.64 (0.53, 0.77) 0.31 (0.27, 0.37)	1.00 0.62 (0.52, 0.76) 0.29 (0.24, 0.34)	1.00 1.86 (1.21, 2.87) 1.55 (1.07, 2.26)	1.00 1.86 (1.20, 2.86) 1.55 (1.07, 2.25)	1.00 0.87 (0.61, 1.23) 0.46 (0.34, 0.63)	1.00 0.86 (0.57, 1.30) 0.41 (0.29, 0.59)
Mother’s age								
15–24 years 25–39 years ≥ 40 years	1.00 0.98 (0.73, 1.32) 1.36 (0.76, 2.44)	1.00 1.01 (0.73, 1.40) 1.58 (0.78, 3.17)	1.00 1.04 (0.93, 1.17) 0.98 (0.78, 1.23)	1.00 1.14 (1.01, 1.29) 1.17 (0.92, 1.49)	N/A	N/A	1.00 0.80 (0.63, 1.01) 0.89 (0.62, 1.29)	1.00 0.81 (0.61, 1.08) 0.85 (0.55, 1.33)
Mother’s education								
None Primary Secondary / higher	1.00 1.11 (0.83, 1.49) 2.29 (1.58, 3.32)	1.00 0.87 (0.61, 1.25) 1.29 (0.81, 2.05)	1.00 1.26 (1.09, 1.46) 2.01 (1.69, 2.40)	1.00 1.12 (0.96, 1.31) 1.75 (1.45, 2.12)	N/A	N/A	1.00 0.84 (0.63, 1.11) 0.79 (0.56, 1.09)	1.00 0.66 (0.46, 0.96) 0.56 (0.35, 0.91)
Household wealth								
Poor Medium Rich	1.00 0.86 (0.49, 1.50) 1.39 (0.79, 2.44)	1.00 0.83 (0.43, 1.61) 0.81 (0.41, 1.60)	1.00 1.41 (1.22, 1.63) 1.56 (1.38, 1.75)	1.00 1.20 (1.03, 1.41) 1.31 (1.14, 1.49)	1.00 0.00 (0.00, 0.00) 1.18 (0.91, 1.53)	1.00 0.00 (0.00, 0.00) 1.18 (0.92, 1.53)	1.00 0.79 (0.61, 1.02) 0.87 (0.71, 1.05)	1.00 0.89 (0.66, 1.20) 1.62 (1.13, 2.32)
Mother’s knowledge								
No Yes	1.00 4.13 (3.05, 5.58)	1.00 4.00 (2.81, 5.68)	1.00 2.66 (2.31, 3.06)	1.00 2.72 (2.35, 3.15)	1.00 0.95 (0.68, 1.32)	1.00 0.96 (0.69, 1.33)	1.00 12.89 (9.87, 16.84)	1.00 14.50 (10.98, 19.17)

UOR, unadjusted odds ratio; AOR, adjusted odds ratio. OR = 1.00 for reference category

^†^ For Sierra Leone mother’s age and education were not available

The majority of children were provided VAS at a health facility in Mozambique (63.9%) and Tanzania (73.4%), while most received their VAS dose at the household in Senegal (47.4%) and Sierra Leone (57.7%) (Table 4). A lack of caregiver awareness about the VAS program was the primary reason for non-supplemented children in Mozambique (52.9%), Senegal (50.1%), and Tanzania (67.8%). In Sierra Leone, multiple reasons for non-supplementation were recorded and the most frequently cited reasons were health workers not coming to the house (42.2%) and the child being absent at the time of the home visit (26.5%). The frequency of VAS refusal was highest in Sierra Leone (7.7%) and < 3% in the other countries (Table 4).

**Table 4:** Location of vitamin A supplementation and reasons for non-supplementation

	Mozambique	Senegal	Sierra Leone ^‡^	Tanzania
Location of VAS receipt Health facility Home Other Did not know	*N* = 722 461 (63.9) 209 (28.9) 49 (6.8) 3 (0.4)	*N* = 3367 1456 (43.2) 1594 (47.4) 313 (9.3) 4 (0.1)	*N* = 3601 1301 (36.1) 2076 (57.7) 217 (6.0) 7 (0.2)	*N* = 1373 1008 (73.4) 210 (15.3) 152 (11.1) 3 (0.2)
Reason for child not receiving VAS ^†^ Not being informed Health worker not coming to house Child absent Child ill Lack of supply Refusal Other Did not know or remember	*N* = 860 455 (52.9) 68 (7.9) 72 (8.4) 4 (0.5) 6 (0.7) 8 (0.9) 59 (6.8) 188 (21.9)	*N* = 3779 1894 (50.1) 939 (24.9) 165 (4.4) 29 (0.8) 46 (1.2) 96 (2.5) 610 (16.1) 0 (0.0)	*N* = 431 96 (22.3) 182 (42.2) 114 (26.5) 3 (0.7) 37 (8.6) 33 (7.7) 26 (6.0) 26 (6.0)	*N* = 1823 1236 (67.8) 60 (3.3) 153 (8.4) 5 (0.3) 35 (1.9) 8 (0.4) 201 (11.0) 125 (6.9)

^†^ Reasons for non-supplementation obtained from caregivers

^‡^ In Sierra Leone multiple reasons for non-supplementation were recorded

## Discussion

In this study, we assessed routine VAS coverage among children under five years in Mozambique, Senegal, and Sierra Leone and campaign-based coverage in Tanzania. Our findings reveal substantial variability in VAS coverage and highlight the fact that routine delivery approaches are not yet achieving the minimum 80% coverage level required for public health impact [3]. The < 50% coverage observed for integrated VAS delivery in Mozambique and Senegal suggests considerable programmatic challenges and contrasts with higher coverage estimates reported for campaigns in SSA [9]. A study involving pooled data from 44 surveys conducted across 13 SSA countries revealed ~ 30% higher VAS coverage through household, as compared to fixed site plus outreach, distribution (91% vs. 63%; *p* < 0.001) and a higher likelihood of VAS among children targeted through a door-to-door supplementation strategy (aOR = 19.0; 95% CI: 17.2, 21.1) [10].

To this end, the < 50% VAS coverage observed for the child health campaign in Tanzania, which typically involves intensive community awareness-raising, was lower than expected and below previously reported administrative and survey-based estimates [11]. However, given that the campaign was facility-based, rather than directly targeting households, we speculate the low coverage was likely due to barriers to bringing children to health facilities or other delivery sites, particularly for caregivers from remote communities. In Mozambique, VAS was less likely for children in rural areas, which suggests the direct and/or indirect costs associated with attending health facilities posed barriers for these communities and or children were missed during outreach visits. Area of residence has shown to be a key factor for VAS in SSA settings, with generally lower uptake in rural areas [12–14]. Therefore, the observed lack of association in the other three countries in our study was unexpected, especially given that the majority of households were located in rural areas more likely to be adversely affected by the shift to non-campaign-based modalities. This variation may be linked to the performance of CHWs who serve as key providers of health information and VAS for remote communities. Evidence suggests the motivation and training of CHWs significantly impact service coverage [15–19], emphasizing the importance of supporting and empowering this important cadre of mobilizers and service providers.

Notwithstanding, our study estimates are consistent with the lower coverage observed during the transition to routine VAS delivery in other regions of Senegal [6] and in Ethiopia [7]. Specific factors likely account for the low routine coverage observed in our study. Well-resourced, and often externally funded, campaigns incorporate intensive training and community mobilization efforts to raise awareness prior to supplementation activities, which are not typically undertaken for routine services operating under tighter budget constraints. Moreover, time, distance, and cost-related challenges associated with care-seeking at health facilities may prevent children from receiving their regular six-monthly VAS doses, in contrast to door-to-door supplementation for which caregivers and children need only to be present at the household.

The substantially higher VAS coverage observed in Sierra Leone, including ~ 90% coverage in rural areas, is primarily attributed to an ‘intensification’ event, in which a substantial proportion of children received VAS at home, which is not typically part of routine VAS programs. Our findings are consistent with other survey-based estimates indicating high VAS coverage in Sierra Leone [20, 21]. In addition, as most caregivers reported health workers not coming to the house to administer VAS as the reason for non-supplementation, communities were likely still sensitized to VAS being delivered on an outreach basis in targeted areas. As household wealth and area were not associated with VAS in Sierra Leone, the higher coverage is likely due to the outreach component, with the cost and distance burdens of seeking supplementation less relevant in these areas and children from poorer and more remote households effectively captured. Evidence from SSA has shown high door-to-door coverage in rural areas and across wealth strata, presumably due to fewer barriers to having children supplemented at home, as opposed to an off-site location [10].

In contrast, maternal education and household wealth were positively associated with child VAS in Senegal and children in higher wealth households were more likely to receive VAS in Tanzania. These findings have been observed in other SSA settings [22–27]. In a multi-country analysis of data from Demographic and Health Surveys, Raut et al. [25] observed maternal education and household wealth were positively associated with child VAS in Ethiopia, Kenya, Nigeria, Senegal, and Tanzania. Other evidence from SSA indicates the importance of caregiver education in fixed site VAS delivery, with little or no education posing a barrier to accessing health-related information and to care-seeking, given the generally poorer living conditions of under-educated populations [10]. Consequently, the lower VAS uptake for children of educated mothers in Tanzania in our study is surprising. We are unable to speculate on this finding, apart from the possibility that these mothers had other obligations preventing them from bringing their children to the health facility or other designated delivery site.

Our study identified age-specific coverage differences, with lower VAS uptake among children ≥ 2 years of age in Mozambique, Senegal, and Tanzania. This is consistent with studies conducted in other regions of Senegal [6] and in Ethiopia [7] highlighting lower routine coverage for older children and may be attributed to the challenges in bringing older children to health centers and/or possibly more frequent visits for infants and younger children for immunizations. In a qualitative study exploring VAS perceptions in Ethiopia, mothers described the burden of bringing multiple under-five children to the health center and the particular challenges of toddlers being too heavy to carry and too young to walk long distances to health facilities [7]. Conversely, in Sierra Leone, children 12–23 months and ≥ 2 years of age were more likely to receive VAS than younger infants which suggests greater reachability for older children through the outreach component of the program in this setting. This was not unexpected as health-seeking behaviors following completion of the childhood immunization schedule are likely to be influenced by socio-demographic factors affecting access to health facilities.

Maternal knowledge of the VAS program was a key determinant of VAS uptake in Mozambique, Senegal, and Tanzania and not being informed was the main reason reported for non-supplementation in these settings. This has been widely observed in other SSA settings [22, 24, 27], emphasizing the importance of health education and community awareness. Multi-country evidence from SSA has shown prior caregiver awareness about supplementation activities to be the strongest predictor of VAS in both door-to-door (OR = 6.8; 95% CI: 5.8, 7.9; *p* < 0.001) and fixed site (OR = 72.5; 95% CI: 66.6, 78.8; *p* < 0.001) delivery models [10]. However, as illustrated by the experience in Sierra Leone where VAS coverage was ~ 90% for children of mothers with and without program knowledge, community awareness may not be as relevant where outreach occurs and rather more important in areas where obtaining VAS requires active care-seeking at health facilities or other sites. In a study conducted in Côte d’Ivoire [28], mobile message reminders to mothers increased facility visits for child VAS and should be explored as a medium for disseminating VAS and other child health information in settings where household mobile phone ownership is high. In addition, fairly remunerated CHWs and other health extension agents may represent an underutilized resource for communicating VAS-related information to increase coverage. Lastly, caregiver/family refusal was not a barrier to VAS in any of the four countries, which is consistent with generally high community acceptance and positive attitudes towards VAS in the SSA context. This suggests higher coverage could potentially be achieved in areas with informed communities and well-functioning delivery systems.

Key strengths of our study include the large samples from four countries representing diverse socio-demographic contexts and the standardized methodology and data collection instruments employed in all countries. However, varying assessment and recall periods may have influenced VAS coverage estimates. Caregiver accounts of children’s VAS status may have been impacted by the extended recall period, in contrast to post-campaign surveys which are typically conducted within one month following supplementation activities. Future studies may benefit from cross-verification of VAS status reported at the household with health facility records. Given the cross-sectional survey design, causal inferences cannot be made for reported associations. Finally, limited data obviated examining the effects of maternal age and education on child VAS in Sierra Leone and other factors not explored may have influenced VAS uptake in our study settings. The cross-country variations in program modalities, health system barriers, and community characteristics are important considerations. The impact of any remaining COVID-19-related program disruptions was not investigated. Lastly, our coverage estimates are for single-dose VAS receipt and, therefore, should not be interpreted to represent full (two-dose) VAS coverage.

## Conclusions

Our findings provide valuable insights into the challenges and complexities of achieving and maintaining high routine VAS coverage in resource-poor settings. While campaigns have been the primary approach in SSA, due to funding constraints, many countries have had to phase-out campaigns and integrate VAS into routine health services. The large gaps in VAS coverage observed in our study settings highlight the critical ongoing need for robust data on the feasibility and effectiveness of innovative approaches to deliver VAS, including using CHWs to provide supplements to remote communities and combining fixed and outreach delivery mechanisms. Strengthening awareness-raising efforts where accessing VAS at health facilities may be particularly problematic should be considered. However, in settings where facilities lack sufficient capacity, maintaining campaigns may be the only option until health systems can be strengthened. More robust evidence is needed to compare the resource intensity of integrated platforms, hybrid models, and campaigns to inform optimal VAS delivery strategies and achieve greater equity across different contexts. Our findings contribute to understanding key elements of successful integrated VAS delivery in the African context. As countries move towards incorporating VAS into routine health services, the essentiality of informed communities and potential losses for older children and socio-economically disadvantaged populations must be considered in policies and programs to sustain the progress made in providing life-saving vitamin A supplements to young children in sub-Saharan Africa.

## Data Availability

The datasets used and/or analysed during the current study are available from the corresponding author on reasonable request.
